# Pharmacogenomics

**Published:** 2008-10

**073**

**A non-protein toxin (NK-NPT1) from Indian *Naja kaouthia* venom arrested cancer cell growth by apoptosis involving caspase 9**

Saha A, Vedasiromoni JR, Gomes A

**Indian Institute of Chemical Biology, Kolkata, India.**

**Introduction:**

Present communication reports purification/characterization of a non-protein cytotoxin from NKV, evaluating its effect on cancer cells.

**Methods:**

Gel filtration, HPLC of NKV for purification of NK-NPT1. Characterization done by NMR and ESI-MS. Evaluation of antiproliferative activity against EAC cells *in vivo* and human leukemic cells (U937, K562) *in vitro* by cell count, MTT assay, morphometric studies, comet assay, annexin-V/PI binding and cell cycle, Caspase 3 and 9 studies. Effect on normal human lymphocytes was studied. Data expressed as Mean±SEM. Statistical analysis with Student’s t-test.

**Results:**

Polyhydroxylated, non-aromatic 452.16D toxin NK-NPT1 purified from NKV, was cytotoxic to EAC cells in mice and human leukemic cells U937 and K562. NK-NPT1 (1mg/kg, i.p/day × 10 days) lowered EAC cell count, cell viability and increased survival time of EAC bearing mice significantly compared to untreated EAC bearing control. Reduced count and MTT values after NK-NPT1 treatment indicated antiproliferative action. IC50 was 33μg/ml for U937/K562 cells. SEM and fluorescence microscopy of NK-NPT1 treated cells showed apoptotic features (apoptotic body formation, nuclear fragmentation and membrane blebbing). Apoptosis was confirmed by phosphatidylserine externalization observed using annexin-V FITC/PI staining and DNA-fragmentation by Comet Assay. NK-NPT1 induced apoptosis by G1 phase arrest of cell cycle via caspase-9 and caspase-3 pathways. Caspase-9 inhibitor prevented apoptosis. Cytotoxicity on normal lymphocyte was lower than that on leukemic cells.

**Conclusion:**

NK-NPT1 is cytotoxic and inhibited cancer cell proliferation (*in vivo/in vitro*) by inducing apoptosis involving Caspase 9 dependent Caspase 3 upregulation. It showed preferential specificity for cancer cells than for normal lymphocytes

**074**

**890E2824: A novel non-redox, non-chelating, competitive inhibitor of 5-lipoxygenase**

Srivastava PK, Gupta S, Shirumalla RK, Dastidar SG, Ray A, Verma AK, Cliffe I

**New Drug Discovery Research, Ranbaxy Research Laboratories, Gurgaon, Haryana, India.**

5- Lipoxygenase (5-LO) is key enzyme in arachidonic acid metabolism, leading to generation of leukotrienes and cysteinyl leukotrienes which are potent chemo-attractant and bronchoconstrictor molecules, respectively. 5-LO is a validated drug discovery target for chronic inflammatory disease like bronchial asthma and USFDA has approved one 5-LO inhibitor, Zileuton, for human use in moderate to severe bronchial asthma. However, use of Zileuton is compromised because of its poor pharmacokinetic property, its tendency to elevate liver enzyme levels and iron chelating capacity. Attempts to move away from Zileuton like molecules (N-hydroxy urea) have not been very successful as most of these inhibitors did not exhibit efficacy in severe bronchial asthma patients while the others were sensitive to redox status of their reaction mechanism. Main objective of the present work is to develop a non-redox, non-iron chelating, competitive inhibitor of 5-LO for Asthma and alternate indications like atherosclerosis. 890E2824 has been found to be a competitive inhibitor of 5-LO with IC_50_ of 438 nM and 325 nm in cell free and cell-based assays, respectively and shows good to moderate selectivity against 12- LO and 15-LO enzymes. This inhibitor retains its activity in high peroxide tone, which mimics the chronic inflammatory conditions and shows efficacy in *ex-vivo* models of inflammation.

**075**

**Anticancer and antiinflammatory activity of chemically modified lupeol derivatives**

J Alex, KK Srinivasan, N Gopalankutty, M Sudheer

**Manipal College of Pharmaceutical Sciences, Manipal University, Manipal, India.**

Lupeol is a biologically active lupane type pentacyclic triterpenoid which exist widely in plants. Lupeol reported to have anticancer, antiinflammatory, antibacterial, antifungal and antimalarial activity. However, in many cases the potency of this compound was relatively weak. Therefore, anticipating highly potent novel structures, synthetic modifications of lupeol was planned. Lupeol was isolated from petroleum ether extract of dried stem bark of Crataeva nurvula. Lupeol (1) on benzoylation gave 3-O- benzoyl derivative (2). Acylation of lupeol with succinic anhydride furnish 3-O- succinyl derivative (3). Lupeol on refluxing with ethyl chloro acetate in 1, 4-dioxane afford Ethyl-lup-20(29)-en-3β-O-acetate (4). Lupeol on two step tosylation elimination reaction yields a 2, 3 dehydro derivative (5). Lupeol was oxidized to 3-oxo derivative (6). 3-oxo derivative (lupenone) on treatment with Phenyl selenyl chloride in ethyl acetate and sequential addition of 30%H_2_O_2_ affords 3-oxo-1-ene derivative (7). Lupenone (6) was further derivatised to 1, 3-oxathiolane derivative (8) by reaction with mercaptoethanol in diethyl ether in the presence of boron trifluoride etherate. Condensation of lupenone with different aromatic aldehydes yields corresponding 2-arylidene derivatives 9a-9d. Cyclisation of arylidene derivatives using hydrazine hydrate gave pyrazoline derivatives 10a-10d. Lupeol and its chemically modified derivatives were characterized and evaluated for their anticancer activity on human cervical cancer cell lines (HeLa) by MTT and SRB assay. Lupeol significantly caused cytotoxicity to cancer cell lines (IC_50_31.5±0.8 μg/ml). 3-oxo-1-ene derivative (7) (IC_50_ 5.5±1.11μg/ml) and pyrazoline derivatives (10aand10b) showed excellent cytotoxic effect (IC_50_12.5±0.95 and10.7±1.21μg/ml respectively) which were comparable with that of the standard cisplatin (IC_50_ 6.58±0.81μg/ ml). Antiinflammatory activities of lupeol and chemically modified compounds were also tested on rats by carrageenan induced rat paw oedema method. Among them, lupeol derivatives 3, 7, 10b and 10d showed excellent antiinflammatory activity comparable to that of standard drug ibuprofen and were found to be more potent than lupeol. Results indicate that chemical modifications of lupeol were useful in improving their potency as anticancer and antiinflammatory agents.

**076**

**Tamarix gallica- a promising hepatoprotective drug**

Chaturvedi S^1^, Tabassum N^2^, Aggrawal SS^3^, Goel N^1^

**^1^MSIP, GGSIPU, Delhi, ^2^University of Kashmir, Srinagar, ^3^DIPSAR, New Delhi, India.**

**Introduction:**

Tamarix gallica, commonly known as Jhavuka in Hindi, has been effectively used in folk medicines as a diuretic, astringent, laxative and in the treatment of diarrhea and dysentery. However, there is still lack of sufficient scientific proof about the hepatoprotective capability. This study is aimed at investigating the role of 50% ethanolic extract of Tamarix gallica leaves against acetaminophen induced hepatocellular damage in albino mice.

**Materials and Methods:**

Albino mice were divided into different groups of eight mice each. Single oral dose of acetaminophen, as a saturated solution in 0.9% saline at the rate of 500mg/kg body weight, was used to induce hepatotoxicity in these mice.

**Result:**

Significant increase in ALT levels, degenerative changes, focal necrosis and sinusoidal dilatation in the liver sections was observed after administration of acetaminophen. Tamarix gallica extract administered at different doses of 25mg, 50mg, 100mg/100g/ day revealed presence of binucleated cells, anisonucleosis, anisocytosis and regenerative changes in the liver.

**Conclusion:**

It can be concluded that extract of leaves of Tamarix gallica showed evidence of antihepatotoxic activity in the mice and this activity was not dose dependent.

**077**

**Development and validation of cassette dosing *(n-in-one)* technique to study the trans-corneal penetration congeneric drugs used in ophthalmic**

Sharma C, Velpandian T, Biswas NR, Saxena R, Nayak N, Ghose S

**All India Institute of Medical Sciences, Ansari Nagar New Delhi – 110 029, India.**

**Introduction:**

The purpose of this study was to evaluate cassette dosing approach in trans-corneal penetration using derivatives of fluroquinolones (FQs) as model group.

**Methods:**

Nine FQs were taken and divided into two groups randomly. Group I comprises of ofloxacin, sparfloxacin, pefloxacin and gatifloxacin. Group II comprises of norfloxacin, lomefloxacin, levofloxacin, ciprofloxacin and moxifloxacin. All the molecules in each group were dissolved in sterile 1.9% boric acid to achieve individual concentration of 0.1%. 50μl of above sterile formulation was instilled on rabbit cornea (N=4). The technique was validated at different drop size volume (12.5μl and 25 μl), different concentration (0.25% and 0.1%) and different pH (4.5, 7.0 and 8.0). Aqueous humor was aspirated through paracentesis after 5, 15, 30, 60, 120 and 240min instillation. All samples were subjected for quantification using HPLC-PDA and LC/MS/MS. A single run method was optimized to co-elute 4-5 fluoroquinolones together.

**Results:**

All drugs showed individualized concentration in aqueous humor following typical pharmacokinetic pattern. Among all drugs, pefloxacin in group I and moxifloxacin in group II showed maximum penetration. The percentage Cmax in group I was found to be 1.57%, 2.64%, 3.2% and 2.32% whereas in group II it was 0.95%, 0.52%, 0.11% and 1.01% respectively

**Conclusion:**

Cassette dosing showed predictable trans-corneal penetration of individual compounds. Few structural modifications/inclusions in the fluoroquinolones correlated well with the corneal penetration. This approach is under further investigator to develop ocular specific fluoroquinolones for maximal corneal penetration with extended spectrum of activity.

